# Using Machine Learning to Measure Relatedness Between Genes: A Multi-Features Model

**DOI:** 10.1038/s41598-019-40780-7

**Published:** 2019-03-12

**Authors:** Yan Wang, Sen Yang, Jing Zhao, Wei Du, Yanchun Liang, Cankun Wang, Fengfeng Zhou, Yuan Tian, Qin Ma

**Affiliations:** 1 Key Laboratory of Symbol Computation and Knowledge Engineering of Ministry of Education, College of Computer Science and Technology, Jilin University, Changchun, 130012 China; 2 Population Health Group, Sanford Research, Sioux Falls, SD 57104 USA; 3 Department of Internal Medicine, Sanford School of Medicine, University of South Dakota, Sioux Falls, SD 57105 USA; 4 Zhuhai Laboratory of Key Laboratory of Symbol Computation and Knowledge Engineering of Ministry of Education, Department of Computer Science and Technology, Zhuhai College of Jilin University, Zhuhai, 519041 China; 5 Bioinformatics and Mathematical Biosciences Lab, Department of Agronomy, Horticulture, and Plant Science, Department of Mathematics and Statistics, South Dakota State University, Brookings, SD 57006 USA; 6 School of Artificial Intelligence, Jilin University, Changchun, 130012 China; 7 Department of Biomedical Informatics, College of Medicine, The Ohio State University, Columbus, OH 43210 USA

## Abstract

Measuring conditional relatedness between a pair of genes is a fundamental technique and still a significant challenge in computational biology. Such relatedness can be assessed by gene expression similarities while suffering high false discovery rates. Meanwhile, other types of features, e.g., prior-knowledge based similarities, is only viable for measuring global relatedness. In this paper, we propose a novel machine learning model, named Multi-Features Relatedness (MFR), for accurately measuring conditional relatedness between a pair of genes by incorporating expression similarities with prior-knowledge based similarities in an assessment criterion. MFR is used to predict gene-gene interactions extracted from the COXPRESdb, KEGG, HPRD, and TRRUST databases by the 10-fold cross validation and test verification, and to identify gene-gene interactions collected from the GeneFriends and DIP databases for further verification. The results show that MFR achieves the highest area under curve (AUC) values for identifying gene-gene interactions in the development, test, and DIP datasets. Specifically, it obtains an improvement of 1.1% on average of precision for detecting gene pairs with both high expression similarities and high prior-knowledge based similarities in all datasets, comparing to other linear models and coexpression analysis methods. Regarding cancer gene networks construction and gene function prediction, MFR also obtains the results with more biological significances and higher average prediction accuracy, than other compared models and methods. A website of the MFR model and relevant datasets can be accessed from http://bmbl.sdstate.edu/MFR.

## Introduction

Biological functions of a gene are cooperating with others when they are in a common cellular environment or the same pathway. Measuring relatedness between a pair of genes is increasingly crucial for understanding the underlying complex interactions and functional relationships of a biological system. Measured relatedness between a pair of genes has been routinely used to construct biological networks^1–5^ and to predict novel genomic functions^6–8^. The gene-gene interaction is usually modeled as a 0/1 (non-interacting/interacting) binary relation between a pair of genes, while the relatedness implies a degree of the relationship between a pair of genes.

The relatedness can be measured by two types of features: expression similarities and prior-knowledge based similarities. The first type of features is usually used to measure the conditional relatedness that is the coexpression level between a pair of genes under a certain condition, such as in inflammation or tumor tissues, according to the correlation between their expression patterns^9–15^, including but not limited to, Pearson correlation coefficient (PCC)^16^, Spearman rank correlation (SRC)^17^, mutual information (MI)^18–21^, partial Pearson correlation (PPC)^22–24^, and conditional mutual information (CMI)^25^. Several coexpression databases have been constructed based on a wild range of available expression data, *e.g*., the COXPRESdb^26^ and the GeneFriends^27^. The second type of features is usually used to measure gene-gene relatedness using the documented biological data and functional annotations in public domain^28–30^, *e.g*., the gene function database Gene Ontology (GO)^31^, the homologous gene database orthoDB^32^, the biological pathway databases KEGG^33^ and Reactome^34,35^, the protein-protein interaction (PPI) databases HPRD^36^ and DIP^37^, and the transcriptional regulatory databases HTRIdb^38^ and TRRUST^39^.

However, there is still a considerable room for improvement of accuracy and robustness in measuring gene-gene conditional relatedness by expression similarities and prior-knowledge based similarities, respectively. First, the accuracy of using expression similarities need to be improved. For example, PCC is known to have a high false discovery rate, especially when the sample size is small, as mentioned in refs^40,41^, which severely impacts the results of further computational analysis and biological interpretations. As there are over 400 million gene pairs in human, a slight increase in false discovery rate would bring an over-estimated number of gene-gene interactions. Second, the robustness of using prior-knowledge based similarities need to be improved as they are only viable for measuring global relatedness^28–30^. Their experiments are usually conducted in a common environment, making prior-knowledge based similarities are not suitable for measuring gene-gene conditional relatedness.

Here, we propose a novel machine learning model, Multi-Features Relatedness (MFR), for measuring conditional relatedness between a pair of genes with an assessment criterion. The goal of MFR is to accurately measure conditional relatedness between genes by integrating expression similarities and prior-knowledge based similarities. Specifically, a gene pair with a low expression similarity will be given a low rank even though they have a high prior-knowledge based similarity, as their relations are not specified under current condition from our point of view; and a gene pair with a high expression similarity and a low prior-knowledge based similarity will also be scored a low rank, as it tends to be a false discovery prediction in coexpression analysis. Gene pairs with both high expression similarities and high prior-knowledge based similarities will be kept and recommended by this model. Intuitively, the problem can be formulated into a single-objective generalized linear logit regression problem under the following hypotheses: (*i*) fitting of relatedness supported by expression similarities is equal to fitting of relatedness supported by prior-knowledge based similarities; (*ii*) both features contribute to fitting on the same level; and (*iii*) the fitting target relatedness are 0/1 (non-interacting/interacting). We use support vector machine (SVM)^42^ with the linear kernel to solve this regression problem and optimize suitable parameters of relevant features. MFR is used to predict gene-gene interactions extracted from the COXPRESdb, KEGG, and TRRUST databases and a benchmark dataset of Pan *et al*.’s study^43^ by the 10-fold cross validation and test verification, and to identify gene-gene interactions collected from the GeneFriends and DIP databases for further verification. The results show that MFR achieves the highest area under curve (AUC) values for identifying gene-gene interactions in the development, test and DIP datasets. Specifically, it obtains an improvement of 1.1% on average of precision for detecting gene pairs with both high expression similarities and high prior-knowledge based similarities in all datasets, comparing to other linear models and coexpression analysis methods. In terms of cancer gene networks construction and gene function prediction, MFR also obtains the results with more biological significances and higher average prediction accuracy than other compared models and methods.

## Materials and Methods

### MFR workflow

**Figure 1:**
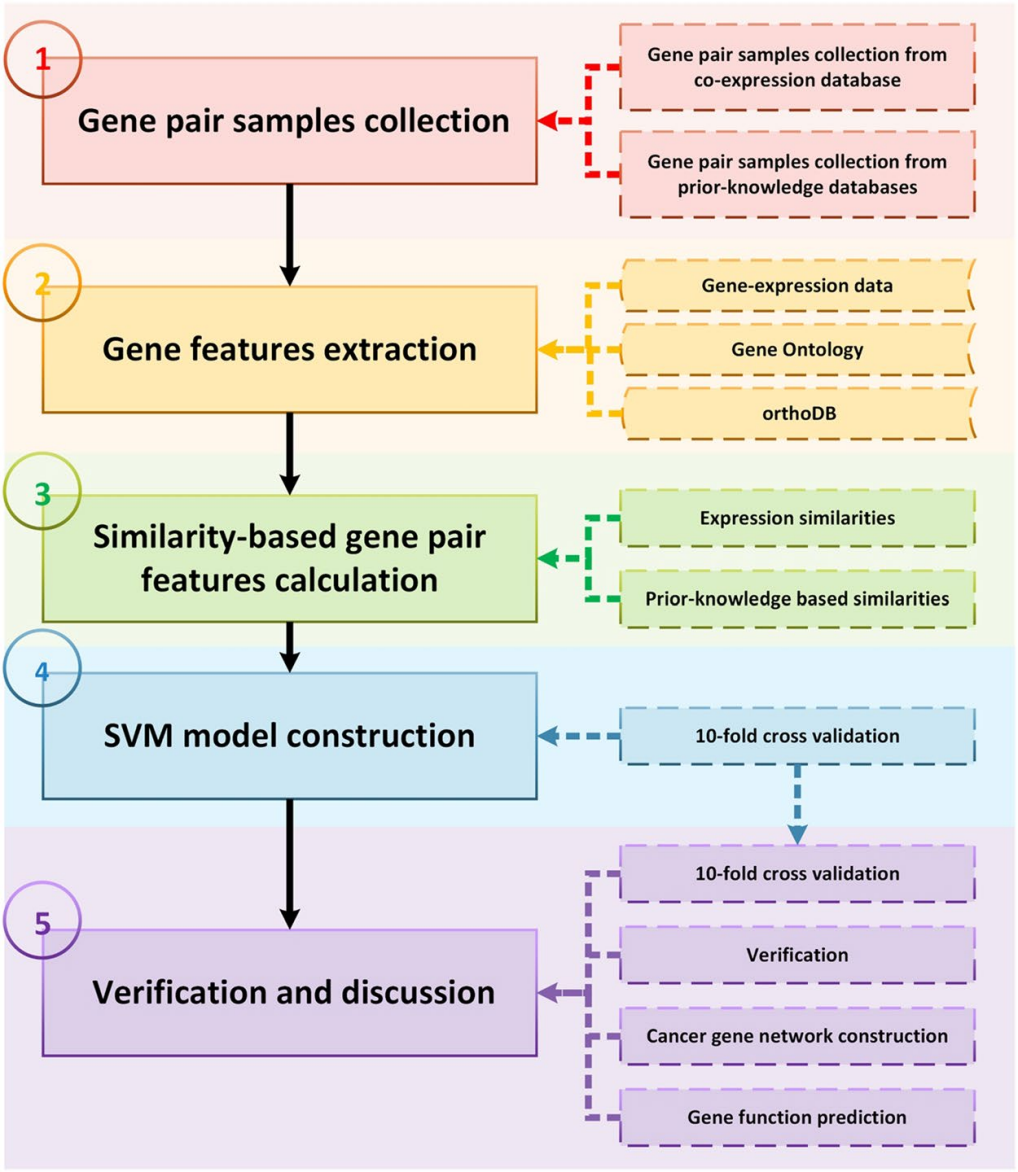
Workflow of MFR model. Five steps are in the workflow, including (*i*) gene pair samples collection, (*ii*) gene features extraction, (*iii*) gene pair features calculation, (*iv*) SVM model construction and (*v*) verification and discussion.

There are five steps in the MFR workflow as shown in Fig. 1: (*i*) gene pair samples collection from the COXPRESDdb, KEGG and TRRUST databases and a benchmark dataset from a published study^43^; (*ii*) gene features extraction from the GEO, GO and orthoDB databases for assessing similarity-based gene pair features; (*iii*) 12 similarity-based gene pair features calculation using four gene features and the Reactome and HTRIdb databases; (*iv*) SVM-based model construction by a 10-fold cross validation, where our model is repeatedly trained by 81% gene pairs and developed by other 9% in 10 times; and (*v*) application of the developed model to detect gene-gene interactions in the remaining 10% gene pairs and the other two verification datasets (the GeneFrineds and DIP datasets), construct cancer gene network, and predict gene functions. The results are compared with other linear models and coexpression analysis methods, including logit regression, linear discriminant analysis (LDA)^44^, PCC, SRC, MI, PPC, and CMI. The trained MFR model is saved as an R data, and the datasets and the results of the current study can be freely downloaded at http://bmbl.sdstate.edu/MFR for academic uses, further verification, and biological analysis.

### Model construction dataset

The gene-pair dataset for MFR model training, development and test is composed of the coexpression and prior-knowledge sub datasets. The former one is retrieved from the COXPRESdb database, where the positives and negatives are the coexpressed and discoexpressed gene pairs, respectively; and the latter one is made up by the KEGG, PPI, and TRRUST sub-sub datasets, where the positives are the gene pairs composed by genes involved in the same pathways, with PPIs or transcriptional regulation relationships, and negatives are the gene pairs composed by genes involved in different pathways, without PPIs and transcriptional regulation relationships. The structure of each sub dataset and sub-sub dataset are listed in Table 1. Some of the negative gene pairs are obtained by permutation of the positives, and then selected randomly to make sure the same number of positives for construction of a model with high generalization. To keep the bias from random permutation and selection, we repeat the process of the dataset generation by 100 times giving rise to 100 datasets. Each of these datasets is used to train, develop and test, and the average AUC value and positive predictive value (PPV) are calculated to develop suitable hyperparameters and compared to other models or methods, where the training, development and test sets are obtained according to the detailed proportion of the sub and sub-sub datasets. In each of the 100 datasets, we obtain 67,000 positive gene pairs and 74,560 negative gene pairs. It notices that the numbers of the positive and negative gene pairs are counted after remove the gene pairs without enough gene-pair features. Also, the fitting target MFR values for the positive gene pairs are marked as 1 s and those for the negatives as 0 s. The detailed information can be found in the following sub-sections.

**Table 1:** Structure of MFR dataset.

sub dataset	Coexpression		Prior-knowledge based					
sub-sub dataset			KEGG		PPI		TURRUST	
Resource database	The COXPRESdb^26^		The KEGG^33^		Ref.^43^		The TRRUST^39^	
Type of gene pair	Positive	Negative	Positive	Negative	Positive	Negative	Positive	Negative
Sample size	30,353	29,607	13,386	13,386	18,227	26,533	5,034	5,034

#### The coexpression sub dataset

In the COXPRESdb database, PCC for each gene pair is transferred to the Mutual Rank (MR) value^45^. The smaller of an MR value, the higher coexpression intensity of the corresponding gene pairs have, and the coexpressed genes of a specific target gene are ranked by their MR values in increasing order. For each gene, we select the first 50 genes in its coexpressed gene list to compose 50 coexpressed gene pairs from the Hsa-m.c4-1 and Mmu-m.c3-1 datasets, respectively. Then the commonly coexpressed gene pairs in both datasets are used as the positive gene pairs. In avoid of the imbalance issue between the positive and negative gene pairs, we select 80 genes in the middle of the coexpressed gene list for each gene to compose 80 discoexpressed gene pairs from the Hsa-m.c4-1 and Mmu-m.c3-1 datasets, respectively. Then the commonly discoexpressed gene pairs in both datasets are used as the negative gene pairs, where PCC of each negative gene pair is around 0. In total, there are 30,353 positive gene pairs and 29,607 negative gene pairs generated in the coexpression sub dataset.

#### The prior-knowledge sub dataset

The prior-knowledge sub dataset is composed by the KEGG, PPI, and TRRUST sub-sub datasets, and the collection of gene pairs are listed as follows.

(A) The KEGG sub-sub dataset. The genes and pathways in metabolism, genetic information processing, environmental information processing, and cellular processes are downloaded from the KEGG database. The 13,386 positive gene pairs are composed by the genes involved in at least three same KEGG pathways, and the 13,386 negatives are randomly selected gene pairs composed by the genes involved in different KEGG pathways to keep the balanced number between the positive and the negative gene pairs.

(B) The PPI sub-sub dataset. This dataset is collected from the study^43^, which has been used as the standard test set for PPI prediction^46–48^, as its reasonable sampling and the balanced number between the positive and negative gene pairs. The 18,227 positive gene pairs are the ones with PPIs from the HPRD database, and the 26,533 negatives are composed of genes located in different organelles, in addition to those gene pairs without PPIs proved by experiments, which are collected from the Negatome database^49^.

(C) The TRRUST sub-sub dataset. The 5,034 gene pairs with transcriptional regulatory relationships from the TRRUST dataset are used as the positive gene pairs. Then we randomly permutate transcription-factor genes with regulated genes as the negative gene pairs, making sure to obtain 5,034 negatives as the same number as the positives.

### Gene features

MFR uses 12 similarity-based gene pair features to assess conditional relatedness between a pair of genes. Ten out of these 12 features are calculated using four gene features, including gene-expression level, GO annotation, homologous profile, and subcellular localization. More details are listed as follows.

#### Expression data

Six hundred two datasets with 15,679 samples from the GEO database^50^ based on the unique Affymetrix Human Genome U133 Plus 2.0 Array platform (released on Dec. 2017) are used as expression data source. Then the pre-processing steps are executed, including log2 scale and quantile normalization. After removing genes without the UniProt IDs^51^, 16,391 protein-coding genes in human are retained for further expression data analysis.

#### Gene ontology data

The GO annotations for human genes are obtained from the GO database (435,975 annotations released on Dec. 2017). Only 43,340 biological process GO terms with experimental evidence are used as functional annotations for genes in our study. The structure of these GO terms can be described as a tree, where the relationships among GO terms fall into four categories: “is a”, “part of”, “has part” and “regulates”. However, we only use 456,781 “is a” relations to assess the GO similarity between genes

#### Homologous data

Over 22 million genes from over 5,000 species, including 169,376 human homologous genes from 20 species, are used to construct the homologous profile data by the orthoDB database (version 9.1).

#### Subcellular localization data

A total of 160,537 cellular component annotations of human genes from the GO database (released on Dec. 2017) are used as the subcellular source to measure subcellular localization similarity between a pair of genes.

### Verification and discussion resources

Besides a test verification, we compare MFR with other linear models (logit regression, LDA) and coexpression analysis methods (PCC, SRC, MI, PPC, and CMI), regarding performances in further verification for the GeneFriends and DIP datasets, construction of cancer gene network, and prediction of KEGG metabolomic gene functions. These resources are described as follows.

#### The GeneFriends and DIP datasets

With the elimination of gene pairs without enough gene-pair features, overall 9,146 coexpressed gene pairs with top 20 PCC values for each gene from the GeneFriends database are used as the positive gene pairs. Considering real coexpressed gene pairs are rare in the whole human genome, the 9,146 randomly selected negative gene pairs generated by permutation of the first and the second genes in the positive gene pairs. Similarly, a total of 1,489 gene pairs with the direct-PPIs from the DIP database (leased on Dec. 2017) are used as the positive gene pairs. The negative gene pairs are 1,489 randomly selected gene pairs composed by permutating the first and the second genes in positive gene pairs. Because the negative gene pairs in the GeneFriends and DIP datasets are both generated by random permutation and selection. To avoid the bias of such random sampling, we repeat the whole dataset generation process for 100 times giving rise to 100 GeneFriends and 100 DIP datasets, respectively. The average AUC value and PPV of each of the 100 datasets are used to compare models or methods in verification.

#### Cancer gene-expression data

The RNA-seq data of four cancer types are downloaded from the TCGA database^52^, each having at least ten cancer samples and ten normal samples, with more details showcased in Table 2. Before further analysis, this expression data is pre-processed, including log2 scale and quantile normalization.

**Table 2:** Sample size of RNA-seq data for four cancer types.

Type	Cancer (Samples)	Normal (Samples)
Bladder urothelial carcinoma (BLCA)	408	19
Breast invasive carcinoma (BRCA)	1095	113
Colon adenocarcinoma (COAD)	285	41
Lung adenocarcinoma (LUAD)	515	59

#### KEGG metabolic genes

In total, 1,403 genes of 84 metabolic pathways from the KEGG database are used to compare different models and methods regarding predicting gene functions. Specifically, 100 out of these genes are randomly selected as the genes without any prior knowledge, and then their functions are predicted by analyzing functional annotations of other 1,303 genes. Such a process is repeated for 100 times, and the average prediction rates are used to indicate the capability for gene function prediction.

### Gene pair feature calculation

While traditional coexpression analysis methods use a signal type of features to measure conditional relatedness between genes, MFR uses multi-features including both expression similarities and prior-knowledge based similarities. Twelve similarity-based gene pair features are used in MFR which are defined as follows.

#### Seven features based on expression similarities

We firstly use average expression levels of each gene, *exp1*, and *exp2*, as the first two features for a gene pair. The following five features are a gene pair’s coexpression levels measured by PCC, SRC, PPC, MI, and CMI. PCC is used to measure linear coexpression relationship; SRC and MI are used to measure non-linear coexpression relationship, where different from SRC based on ranks, MI determines how similar the joint distribution of two genes’ expression levels is to the products of factored marginal distribution for indicating the association between their expressions. PPC is used to measure direct linear coexpression, which is the coexpression relationship between a pair of genes measured avoiding any influence of other genes; Similarly, CMI is used to measure direct non-linear coexpression.

#### One feature based on the gene ontology similarity

The GO similarity (*goSim*) is used as the eighth feature because the genes with interaction are considered being involved in the similar biological process. It can be defined as:

1 $$goSi{m}_{i,j}=ma{x}_{o\in {O}_{i},q\in {O}_{j}}\frac{2\times \,\mathrm{log}(Pms(o,q))}{\mathrm{log}(P(o))+\,\mathrm{log}(P(q))}$$

2 $$Pms(o,q)=mi{n}_{c\in A(o,q)}P(c)$$

3 $$P(o)=\,\frac{|D(o)|+1}{|D(root)|+1}$$

where *O*_*i*_ and *O*_*j*_ indicates the GO term sets used for annotating gene *i* and *j*, respectively; *A*(*o*, *q*) is the common ancestor set of GO term *o* and *q; P(o)* is the probability of a gene annotated by an instance of GO term *o*^53^; *D(o)* and *D(root)* indicate the descendant GO term sets of GO term *o*, and the *root* GO term, respectively.

#### One feature based on subcellular localization similarity

The ninth feature, subcellular localization similarity (*lcSim*), is used to calculate the probability for two protein-coding genes appearing in a common organelle. It can be defined as:

4 $$lcSi{m}_{i,j}=\frac{|{L}_{i}\cap {L}_{j}|}{|{L}_{i}\cup {L}_{j}|}$$

where *L*_*i*_ and *L*_*j*_ are the subcellular localization sets of proteins encoded by the genes *i* and *j*, repressively.

#### One feature based on homology similarity

Since common presence and absence of two genes in many species suggest a potential functional relatedness between them, the homology similarity (*hgSim*) is used as the 10th feature calculated using an improved Pearson correlation method^54^ as:

5 $${\rm{hg}}Si{m}_{i,j}=\frac{N\times M-{n}_{i}\times {n}_{j}}{\sqrt{(N\times {n}_{i}-{n}_{i}^{2})\times (N\times {n}_{j}-{n}_{j}^{2})}}$$

where *n*_*i*_ and *n*_*j*_ are the numbers of species whose genome contains the orthologous genes of gene *i* and *j*, respectively; *N* = 21 is the total number of species we use, and *M* is the number of species whose genome contains both orthologous genes of gene *i* and *j*.

#### One feature based on Reactome similarity

Overall, 202,772 gene-gene interactions derived from the Reactome pathways are used to construct an unweighted graph, in which nodes represent genes and edges represent interactions between genes. The normalized distance of a gene pair is used as the 11th feature named as Reactome similarity (*rxSim*), which is defined as:

6 $$rxSi{m}_{i,j}=1-\frac{di{s}_{i,j}}{di{s}_{max}}$$

where *dis*_*i,j*_ is the shortest distance between gene *i* and *j*, and *dis*_*max*_ is the shortest distance between the farthest gene pair in the graph.

#### One feature based on transcriptional regulatory similarity

Totally 284 transcription factors, 18,302 regulated genes, and 51,871 transcriptional regulatory interactions between them are obtained from the HTRIdb database. If there is a record that a gene pair has a transcriptional regulatory interaction, the transcriptional regulatory similarity (*trSim*) used as the 12th feature of this gene pair is 1, otherwise is 0.

### SVM model construction

**Figure 2:**
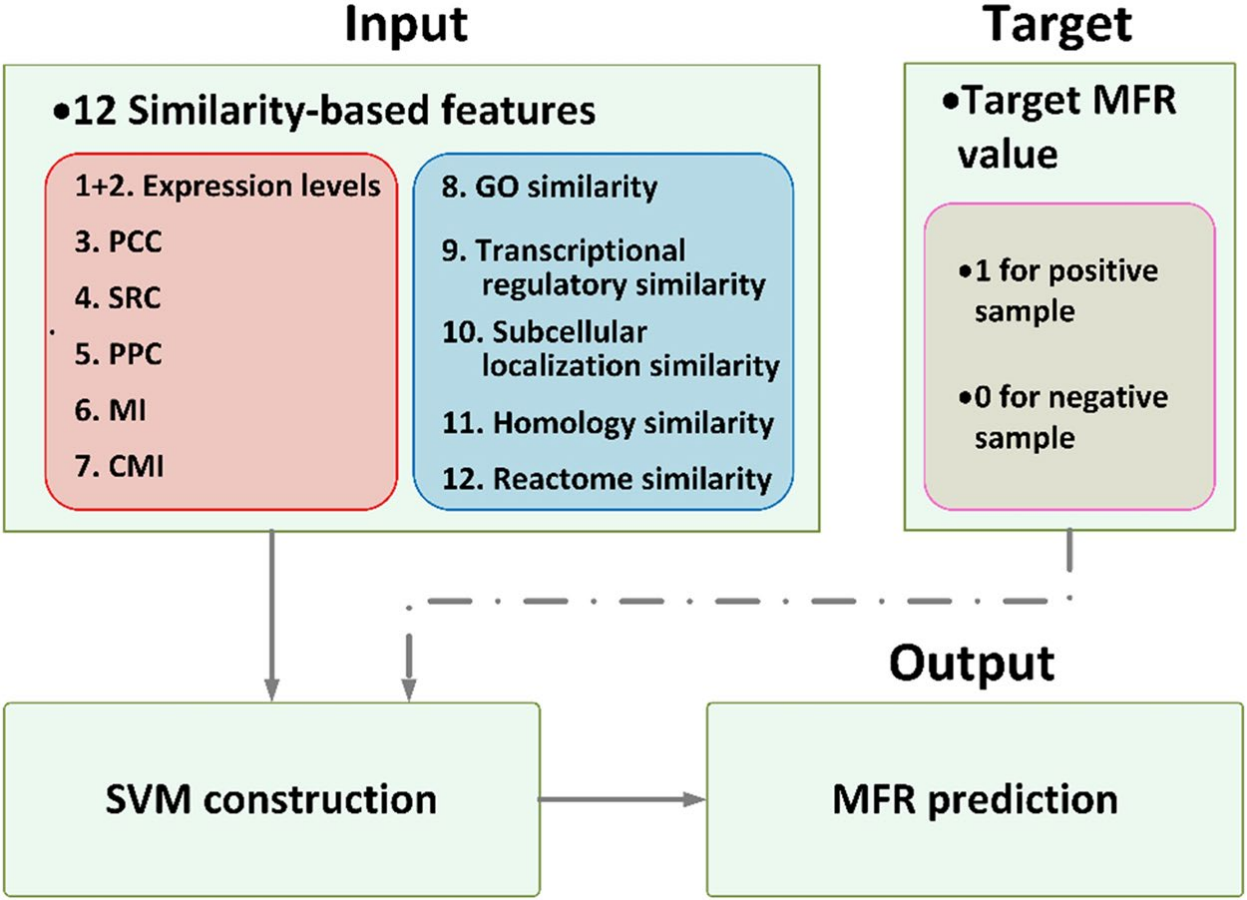
Structure of the MFR model. The model is based on SVM and uses 12 similarity-based gene pair features as input; and the output value, namely MFR, is applied as an assessment criterion for measuring conditional relatedness between genes.

MFR is designed based on SVM, which is a supervised learning model, with associated learning algorithms for classification and regression analysis. The motivation is to classify data by using the best hyperplane that is the one that represents the most extensive separation, or margin, between two classes. We take a total of 12 similarity-based gene pair features as input, and the output value as an assessment criterion, namely MFR, for detecting the conditional relatedness between a pair of genes (see Fig. 2). For model training, we provide the target MFR values (labels) marked as 1 s and 0 s for the positive and the negative gene pairs, respectively. Given ***X*** = {***x***_1_, ***x***_***2***_, *…*, ***x***_*n*_} and ***Y*** = {*y*_1_, *y*_2_, *…*, *y*_*n*_}, where ***x***_*i*_ and *y*_*i*_ indicates the vector of 12 similarity-based gene-pair features and the target MFR value (label) of the *i*th gene pair, repressively, the MFR model can be construction by conduction Formula (7):

7 $$\begin{array}{c}{ma}{{x}}_{\alpha }(\sum _{i=1}^{n}{\alpha }_{i}-\frac{1}{2}\sum _{i,j=1}^{n}{\alpha }_{i}{\alpha }_{j}{y}_{i}{y}_{j}{{\boldsymbol{x}}}_{i}^{T}\cdot {{\boldsymbol{x}}}_{j})\\ s.t.\sum _{i=1}^{n}{\alpha }_{i}{y}_{i}=0;0\le {\alpha }_{i}\le C,i=1,2,\ldots ,n\end{array}$$

where ***α*** = {*α*_1_,*α*_2_, …, *α*_*n*_} indicates Lagrange multipliers, which are solved by SMO (sequential minimal optimization)^55^. Then predicted ${\widehat{MFR}}_{i}$ value of *i*th gene pairs is defined as:

8 $${\widehat{MFR}}_{i}=sigmoid(\sum _{j=1}^{n}{\alpha }_{j}{y}_{j}{{\boldsymbol{x}}}_{j}^{T}\cdot {{\boldsymbol{x}}}_{i}+\hat{b})$$

where $\hat{b}$ indicates the bias defined as ref.^42^.

Because there are not enough positive gene pairs with both high expression similarities and high prior-knowledge based similarities for directly training, we collect positive gene pairs with high expression similarities and the corresponding negatives to compose the coexpression sub dataset. Similarly, we collect positive gene pairs with high priori-knowledge based similarities and the corresponding negatives to compose the priori-knowledgesub dataset. Then MFR is trained by gene pairs in the whole dataset including both coexpression and prior-knowledge sub datasets at the same time to provide our model the capability for identification of gene pairs with both high expression similarities and high prior-knowledge based similarities, rather than trained by coexpression sub dataset or prior-knowledge sub dataset separately. And a higher MFR value indicates that two genes are more likely to be interacting with each other. In detail, we employ LIBSVM^56^ with the linear kernel to implement our model.

**Figure 3:**
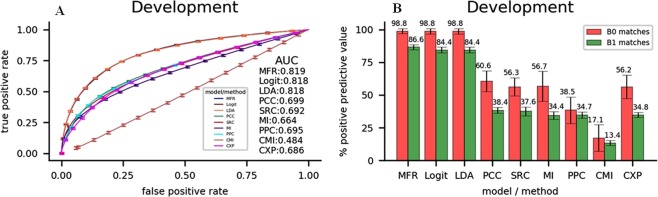
(**A**) ROCs of nine models or methods for identifying gene-gene interactions by the 10-fold cross-validation. (**B**) Average PPVs of nine models or methods for detecting B0/B1 matched gene pairs by 10-fold cross-validation.

**Figure 4:**
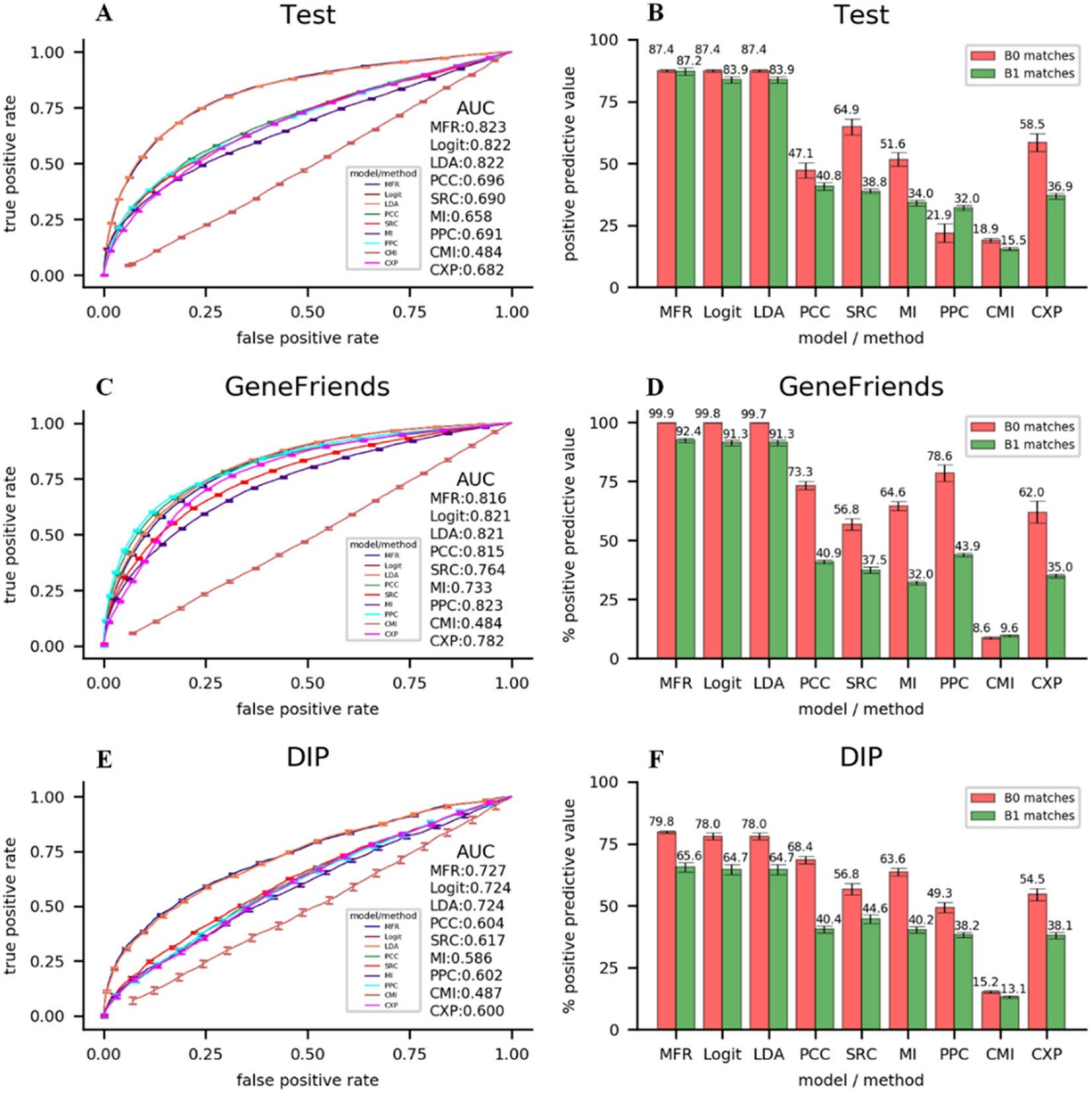
ROCs of nine models or methods for identifying gene-gene interactions in the (**A**) test, (**C**) GeneFriends and (**E**) DIP datasets. Average PPVs of nine models or methods for detecting B0/B1 matched gene pairs in the (**B**) test, (**D**) GeneFriends and (**F**) DIP datasets.

MFR is constructed by the 10-fold cross validation, in which we use 81% of the gene pairs for training and 9% for development. The procedure is repeated by 10 times. The hyperparameters with the highest average AUC value of the whole cross-validation are selected. Then we use the rest 10% gene pairs to conduct test verification. The result of our model in the 10-fold cross validation and test verification is compared with those of other linear models or coexpression analysis methods as shown in Results (see Figs 3 and 4). After training and development, the weight *w*_*S*_ of the gene-pair feature *S* is finalized as *w*_*exp*1_ = −0.810, *w*_*exp*2_ = −0.807, *w*_*PCC*_ = −0.017, *w*_*SRC*_ = 0.840, *w*_*MI*_ = 4.875, *w*_*PPC*_ = 2.414, *w*_*CMI*_ = −0.055, *w*_*goSim*_ = 0.972, *w*_*lcSim*_ = 1.198, *w*_*hgSim*_ = 0.433, *w*_*rxSim*_ = 0.544 and *w*_*trSim*_ = 0.668, indicating MI, PPC, *goSim* and *lcSim* are the most important gene-pair features for MFR model, while PCC and CMI are the least important. The top four important features contain two expression similarities and two priori-knowledge based similarities indicating both kinds of features contribute to accurately measuring relatedness of a pair of genes. MI and PPC obtain the largest weights among expression similarities maybe because, before calculation of MI, the expression levels of genes are discretized according to study^57^, making MI get stronger robustness on the noise of gene expressions, and PPC has more complementarity with MI compared with other expression similarities, as other expression similarities, specially PCC and CMI, have some resemblance with MI^58,59^. The larger weights of *goSim* and *lcSim* than other priori-knowledge based similarities indicate two genes with the related functions and the similar organelle locations mostly have a strong relatedness. The negative weights of *exp1* and *exp2* indicate the punishment of the exorbitant expression, as two of the genes in a gene pair are very hard to have exorbitant expressions both, and the exorbitant expression of a gene usually implies a gap of expression with the other gene, indicating a low relatedness between these genes.

### Performance evaluation

We compare the performances of MFR with other two linear models, *i.e*., logit regression and LDA^44^, and five coexpression analysis methods (PCC, SRC, PPC, MI, and CMI). We choose logit regression and LDA because they are widely used multi-features generalized linear logit regression models^60–63^. And the five coexpression analysis methods are selected since they are traditional methods in measuring conditional relatedness between a pair of genes^16–25^. To make a fair comparison with linear models with multi-features, we also add the sixth coexpression analysis method, so-called CXP, which is the integration of PCC, SRC, PPC, MI, and CMI. Specially, the average value of these five methods is used as the assessment criterion of CXP, comparable with the result from other multi-features methods, such as MFR, logit, and LDA. First, we compare different models and methods in detecting gene-gene interactions on verification datasets using the receiver operating characteristic curve (ROC)^64^, where gene-gene interactions indicate positive gene pairs with high expression similarities or high prior-knowledge based similarities. And we use PPV^65^ to compare different models and methods in identifying gene pairs with both high expression similarities and high prior-knowledge similarities as defined in Section 2.7.2. Then, we conduct pathway enrichment analysis to identify the pathways significantly influenced by the increased glutamine and glutamate metabolism, on gene modules identified in cancer gene networks, where nodes represent up-regulated genes and edges show relatedness measured by each model or method. Finally, the shortest-path method^66^ is applied to predict functions of genes pretending to have no prior knowledge, on the KEGG metabolic gene networks, where nodes represent genes involved in KEGG metabolism pathways and edges represent relatedness calculated using different models and methods, respectively.

#### Receiver operating characteristic curve

The ROC curve with its area under the curve (AUC) is a widely used evaluation tool for performance comparison of different methods. It is made by plotting true positive rate (*TPR*) against false positive rate (*FPR*), which are defined as:

9 $$TPR(n)=\frac{TP(n)}{P}$$

10 $$FPR(n)=\frac{FP(n)}{N}$$

where *TP(n)* indicates the true positive among top *n* ranked gene-gene interactions, *FP(n)* indicates the false positive among top *n* ranked gene-gene interactions, *P* indicates the total number of interacting gene pairs, and *N* indicates the total number of non-interacting gene pairs.

#### Positive predictive value

The positive predictive value (PPV), so-called precision, is an intuitive indicator for evaluating prediction results among models, and a high value of PPV indicates the accuracy of a model. PPV is defined as:

11 $$PPV=\frac{TP}{TP+FP}$$

where *TP* and *FP* are the true positive and the false positive among gene pairs, respectively.

As it is very hard to give a precise definition of a gene pair with both high expression similarities and high prior-knowledge based similarities, we define a gene pair labeled a B0 match if its PCC or SRC values larger than 0.5 and the *goSim* and *lcSim* values larger than 0.5; and labeled a B1 match if its PCC or SRC values larger than 0.3 and the *goSim* and *lcSim* values larger than 0.3. And then the PPV of top 5% ranked gene pairs against B0 matched gene pairs, and the PPV of the top 10% ranked gene pairs against B1 match gene pairs are used to approximately compare models in terms of prediction of gene pairs with both high expression similarities and high prior-knowledge based similarities.

#### Up-regulated genes identification

A gene is identified to be up-regulated if the fold-change between the average expression level in cancer samples and that in normal samples is greater than 1.5 and with a *q*-value < 0.05 measured by the limma *t*-test^67^.

#### Fast greedy modularity optimization method

In the study^68^, a method was proposed to find modules in networks by greedy optimization of modularity^69^. The fast-greedy modularity optimization method^70^ performs the same greedy optimization as the method of^68^, but it runs much faster due to the lower computational cost.

#### Pathway enrichment analysis

Pathway enrichment analysis is conducted over a given set *C* of up-regulated genes against the pathways in KEGG. The statistic significant *p*-value of gene set *C* with *n* genes enriching pathway *P* with *K* genes can be defined as:

12 $$Pvalue(k)=1-\sum _{i=0}^{k-1}\frac{(\begin{array}{c}K\\ i\end{array})(\begin{array}{c}N-K\\ n-i\end{array})}{(\begin{array}{c}N\\ n\end{array})}$$

where *N* = 18,420 is the total number of human genes and *k* is the number of genes in $C\cap P$. Then the *p*-value is adjusted to be a *q*-value to restrict the false discovery rate^71^. And we consider the *C* enriches *P* if *q*-value < 0.01.

#### Shortest-path method

For identifying all the genes with GO annotations on the shortest path, the shortest-path method^66^ is applied to find the lowest common ancestor of their GO annotations. If the ancestor is less than three levels below the root of the GO tree, it is assigned to the genes without any GO annotation on the shortest path as their functions. A gene is labeled a L0 match if one of the predicted GO annotations is its known GO annotation and labeled a L1 match if one of the predicted GO annotations is its known GO annotations’ direct parents^66^. Then L0 and L1 match ratios relative to the total number of genes without any GO annotations are used to compare each model or method regarding gene function prediction.

## Results

### 10-fold cross-validation

We compare the precision of identifying gene-gene interactions by MFR with the other linear models and coexpression analysis methods on the development datasets. The ROC results by the 10-fold cross-validation of different models and methods are showcased in Fig. 3. The linear models including MFR, logit, and LDA are more suitable for detecting gene-gene interactions, as their average values of AUC are all larger than those of coexpression analysis methods. Among these linear models, our model based on SVM performs the best and obtains the largest average AUC value of 0.819. In terms of prediction of the gene pairs with both high expression similarities and high prior-knowledge based similarities, the average PPVs of B0 and B1 matched gene pairs for linear models are also larger than those for coexpression analysis methods, where MFR obtains the best performance for the largest average PPVs of B0 and B1 matched gene pairs of 0.988 and 0.866, respectively.

### Verifications on the test, GeneFriends and DIP datasets

The robustness evaluation is carried out through examining the performances of different models and methods in detecting gene-gene interactions, and in identifying gene pairs with both high expression similarities and high prior-knowledge based similarities on three kinds of verification datasets, including the test datasets, GeneFriends datasets, and DIP datasets. Specially, the results on GeneFriends datasets indicate the robustness in detecting gene-gene interactions and gene pairs from coexpression data, and those on DIP datasets indicate the robustness in identifying gene-gene interactions and gene pairs from prior-knowledge based data. As showcased in Fig. 4, the linear models (MFR, logit, and LDA) are better from the result of verification, as their average AUC values and PPVs are all larger than those of coexpression analysis methods. MFR obtains the largest average AUC values on all verification datasets except the GeneFriends dataset, and the largest average PPVs on all verification datasets, indicating our SVM-based model has the best robustness.

### Cancer gene network construction

**Figure 5:**
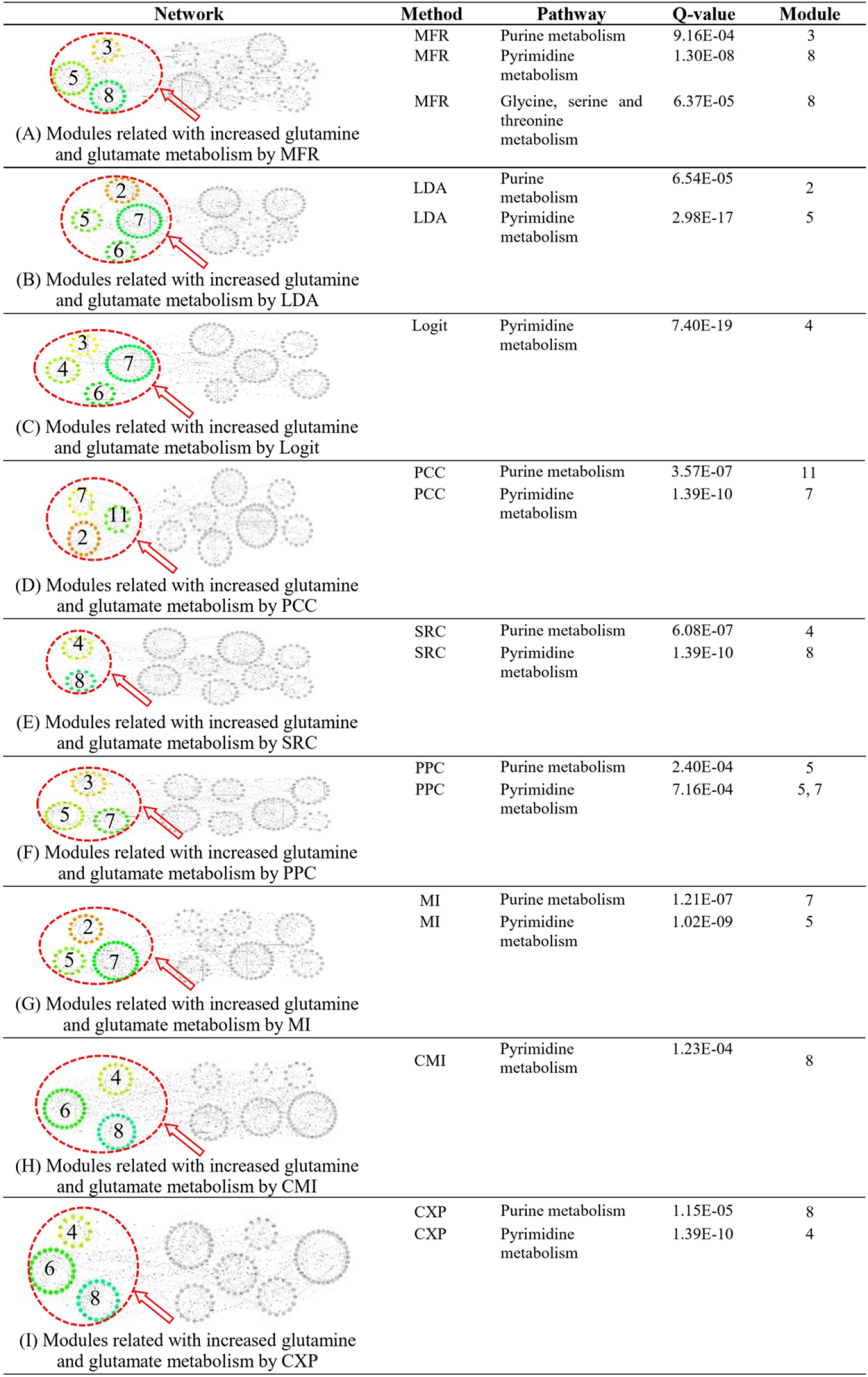
Metabolic pathways are predicted to be directly influenced by increased glutamine and glutamate metabolism in nine BRCA gene networks.

The relatedness between a pair of genes can be used as a similarity between the corresponding nodes in a constructed biological network, where genes in a set of highly interconnected genes (module) tend to be involved with relative biological processes. We utilize this property to predict metabolic pathways significantly influenced by increased glutamine and glutamate metabolism in four cancer types, which are BLAC, BRCA, COAD, and LUAD. Glutamine and glutamate metabolism are reported to be increased in various cancers^72,73^, especially in bladder cancer^74^, breast cancer^75–81^, colon cancer^76,78,79,82^, and lung cancer^76,78,79,83^. They are also considered to be closely related to cancer’s proliferation, invasion, and metastasis^84^. For each cancer type, we measure relatedness between up-regulated metabolic genes using MFR, other linear models, and coexpression analysis methods, respectively. Then the up-regulated metabolic genes and their relatedness in each cancer type are used to construct networks for each model and methods, where nodes represent genes, and two genes are connected if the MR of their relatedness is among top three. We collect 21 genes, including eight rate-limiting enzyme genes for glutaminolysis and 13 genes directly catalyzing reactions of glutamine or glutamate, defined as the gene markers for glutamine and glutamate metabolism (see Table 3), inspired by a recent study^85^. After identifying modules containing up-regulated gene markers, the pathway enrichment analysis is conducted on such modules to predict metabolic pathways directly influenced by increased glutamine and glutamate metabolism, which are the enriched with up-regulated gene markers, as shown in Fig. 5 and Supplement Figures S1–S3.

**Table 3:** Gene markers for glutamine and glutamate metabolism.

Gene	Description	Go Term
ASNS	Asparagine Synthetase	asparagine biosynthetic process
ALDH18A1	Aldehyde Dehydrogenase 18 Family Member A1	proline biosynthetic process
CAD	Carbamoyl-Phosphate Synthetase 2, Aspartate Transcarbamylase, And Dihydroorotase	‘*de novo*’ pyrimidine nucleobase biosynthetic process
CS	Citrate Synthase	tricarboxylic acid cycle
CTPS	CTP Synthase 1	‘*de novo*’ CTP biosynthetic process
CTPS2	CTP Synthase 2	‘*de novo*’ CTP biosynthetic process
DLD	Dermcidin	2-oxoglutarate metabolic process
DLST	Dihydrolipoamide S-Succinyltransferase	tricarboxylic acid cycle
GFPT1	Glutamine-Fructose-6-Phosphate Transaminase 1	UDP-N-acetylglucosamine biosynthetic process
GFPT2	Glutamine-Fructose-6-Phosphate Transaminase 2	UDP-N-acetylglucosamine biosynthetic process
GLUL	Glutamate-Ammonia Ligase	glutamine biosynthetic process
GLS	Glutaminase	glutamate biosynthetic process
GLS2	Glutaminase 2	glutamate biosynthetic process
OGDH	Oxoglutarate Dehydrogenase	tricarboxylic acid cycle
GGDHL	Oxoglutarate Dehydrogenase-like	tricarboxylic acid cycle
PFAS	Phosphoribosylformylglycinamidine Synthase	‘*de novo*’ IMP biosynthetic process
PPAT	Phosphoribosyl Pyrophosphate Amidotransferase	‘*de novo*’ IMP biosynthetic process
PSAT1	Phosphoserine Aminotransferase 1	‘*de novo*’ IMP biosynthetic process
GCLC	Glutamate-Cysteine Ligase Catalytic Subunit	glutathione biosynthetic process
GCLM	Glutamate-Cysteine Ligase Modifier Subunit	glutathione biosynthetic process
GSS	Glutathione Synthetase	glutathione biosynthetic process

**Figure 6:**
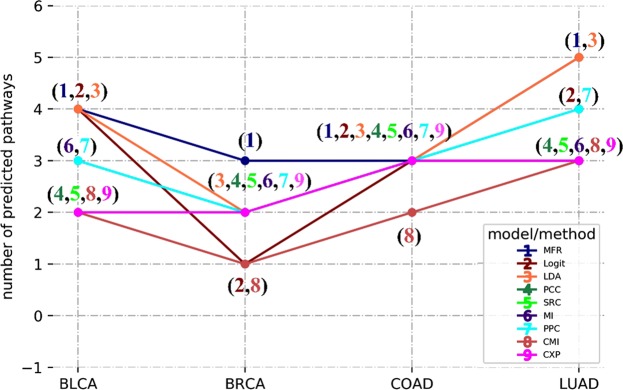
Number of metabolic pathways predicted to be directly influenced by increased glutamine and glutamate metabolism in four cancer types. These pathways were predicted in cancer gene networks, where nodes represent up-regulated metabolic genes and edges represent relatedness between genes, measured by the five linear models and six coexpression analysis methods.

As shown in Fig. 6, we obtain the best prediction results from MFR-based networks. We predict 15 pathways directly influenced by increased glutamine and glutamate metabolism in all four cancer types, which is the most among all the models and methods. For example, in BRCA, there are three pathways are predicted to be directly related to increased glutamine and glutamate metabolism, agreeable with studies^74,86^. However, only one or two of the three pathways are predicted by other models or methods. For MFR, the prediction of the glycine, serine, and threonine metabolism pathway is further confirmed as PSPH (phosphoserine phosphatase) found to be up-regulated in BRCA. Especially, PSPH acts as a rate-limiting enzyme involved in serine synthesis from glutamate^87^.

### Gene function prediction

We randomly select 100 out of 1,403 genes involved in the KEGG metabolism pathways and pretend that there is no prior knowledge with them, and then we predict their functions through analyzing GO annotations of other 1,303 genes. This process repeats for 100 times. First, we use MFR, the other linear models, and coexpression analysis methods to measure the relatedness between each pair from the 1,403 genes, respectively. For each linear model, as selected genes pretend to be without prior knowledge, we mainly use expression similarities (PCC, SRC, MI, PPC, and MI) to calculated their relatedness with other genes, and set other gene-pair features to be 0.5. The relatedness measured by different models and methods are normalized as follow: (*i*) rank the values of each model or method; and (*ii*) for each model or method, replace its values with the corresponding PCC values according to the ranks. Then the 1,403 genes and their relatedness are used to construct gene networks for different models and methods, respectively. In the constructed networks, nodes represent genes, edges represent relatedness between genes, and measured values of relatedness are used as the weights of edges. The edges with weights less than 0.6 are removed based on the procedure in the previous study^66^. Finally, each network contains 1,403 nodes and 14,067 edges.

**Figure 7:**
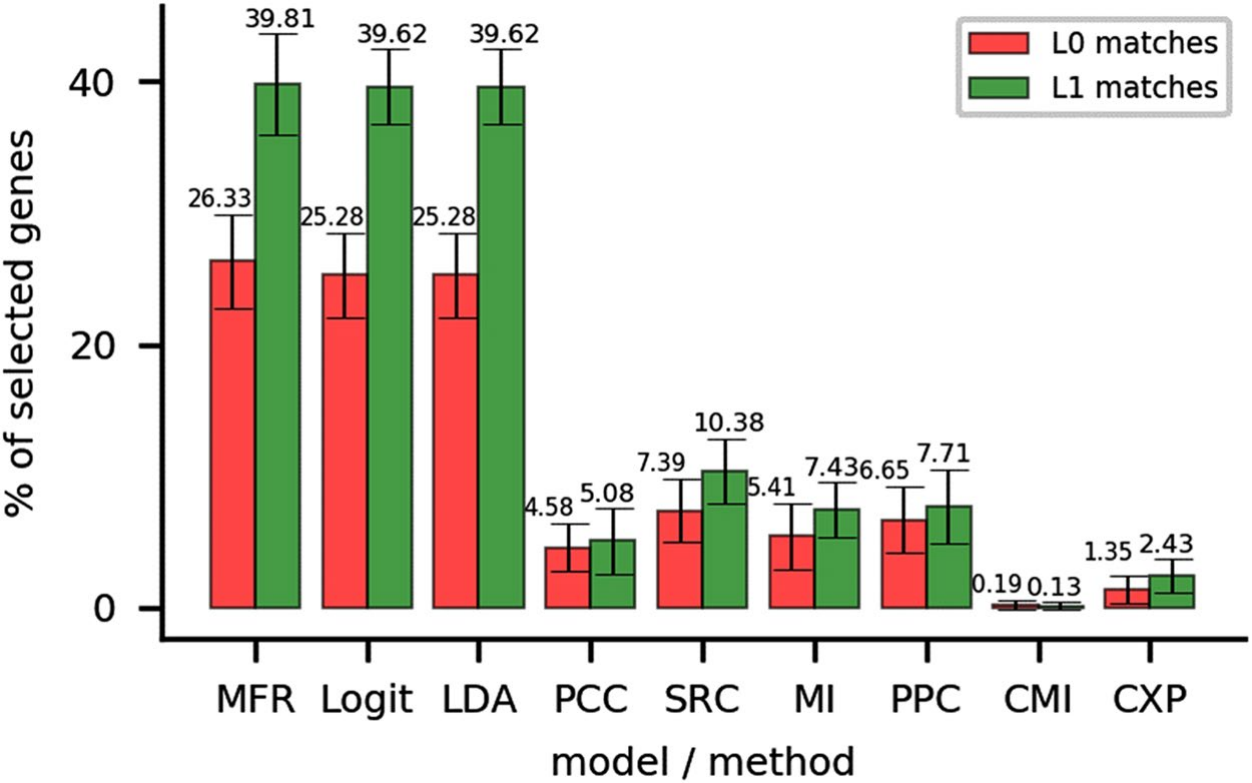
Percentages of L0- and L1-matched selected genes in the nine KEGG metabolic gene networks. In these networks, nodes represent genes involved in KEGG metabolism pathways, and edges represent relatedness between genes, measured by the nine models or methods.

A broadly used shortest-path method is applied to predict the function of selected genes. As shown in Fig. 7, in MFR-based gene network, the shortest-path method achieves notably accurate results, where it successfully calls average 39.81%/26.33% of the selected genes at the L1/L0 levels. However, it only calls average 39.62%/25.28%, 39.62%/25.28%, 5.08%/4.58%, 10.38%/7.39%, 7.43%/5.41%, 7.71%/6.65%, 0.13%/0.19% and 2.43%/1.35% of the selected genes at the L1/L0 levels in logit-regression-, LDA-, PCC-, SRC-, PPC-, MI- CMI- and CXP- based networks, respectively. Overall, the results suggest that MFR outperforms other models and methods regarding gene function prediction, as it constructs better networks on genes with prior knowledge and benefits functional prediction of genes.

## Discussion and Conclusion

In this paper, we propose a novel machine learning model for measuring conditional relatedness between genes, named MFR, by integrating seven expression similarities and five prior-knowledge based similarities. Specifically, gene pairs with both high expression similarities and high prior-knowledge based similarities will be kept and recommended by our model. At first, we conduct the MFR model in 10-fold cross-validation. Then we used the MFR model in a test verification and two further verifications on the GeneFriends and DIP datasets. Finally, the MFR model is used to construct cancer gene networks and predict gene functions. All the results are compared with those of other models or methods (see Table 4).

**Table 4:** Performances of the nine models or methods for different applications.

Application	Evaluation	MFR	Logit	LDA	PCC	SRC	MI	PPC	CMI	CXP
10-fold cross-validation	AUC	**0.819**	0.818	0.818	0.699	0.692	0.664	0.695	0.484	0.686
	B0 + B1	**0.927**	0.916	0.916	0.495	0.469	0.456	0.366	0.152	0.455
Test verification	AUC	**0.823**	0.822	0.822	0.696	0.690	0.658	0.691	0.484	0.682
	B0 + B1	**0.873**	0.856	0.856	0.440	0.518	0.428	0.270	0.172	0.477
GeneFriends verification	AUC	0.816	**0.821**	**0.821**	0.815	0.764	0.733	0.823	0.484	0.782
	B0 + B1	**0.962**	0.957	0.957	0.571	0.471	0.483	0.613	0.091	0.485
DIP verification	AUC	**0.727**	0.724	0.724	0.604	0.617	0.586	0.602	0.487	0.600
	B0 + B1	**0.727**	0.713	0.713	0.544	0.507	0.519	0.438	0.142	0.463
Constructing a cancer gene network	NPP	**15**	12	14	10	10	11	12	8	10
Predicting gene function	L0 + L1	**33.07**	32.45	32.45	4.83	8.89	6.42	7.18	0.16	1.89

B0 + B1 indicates the average value of PPVs of B0- and B1-matched genes; NPP indicates the number of predicted metabolic pathways; L0 + L1 indicates the average number of L0- and L1-matched genes

In terms of identifying gene-gene interactions, multi-features models, such as MFR, logit and LDA performance better than coexpression analysis methods including PCC, SRC, MI, PPC, CMI, and CXP in the 10-fold cross-validation and verifications. Hence, the models integrating both expression similarities and prior-knowledge based similarities can avoid the shortage of using only one kind of expression similarities. And among those multi-feature models, MFR performances best in the 10-fold cross-validation, test verification, and one further verification on DIP datasets (except GeneFriends datasets), indicating the SVM-based model is more suitable for resolving the conflict of fitting relatedness supported by coexpression and those supported by prior knowledge at the same time. It also notices that MFR has better performances in the datasets containing the gene pairs extracted from both coexpression data and prior-knowledge based data (*i.e*., development and test datasets) and datasets containing the gene pairs extracted from prior-knowledge based data (*e.g*., the DIP datasets). On the contrary, logit and LDA models have better performances in the datasets containing the gene pairs extracted from only coexpression data, such as the GeneFriends datasets. In other words, logit and LDA models prefer gene pairs with high expression similarities, comparable with MFR. As a result, MFR is relatively good at detecting gene pairs with both high expression similarities and prior-knowledge based similarities and obtains the best results in all the datasets. For a real biological problem, some of the important gene pairs usually having attributes such as coexpression, like positive gene pairs collected from coexpression data, and the others typically have attributes such as PPI, like positive gene pairs collected from prior-knowledge based data. Additionally, gene pairs with both high expression similarities and high prior-knowledge based similarities are more likely the real important interacting gene pairs. So MFR is more suitable for practical applications, such as biological network construction and genomic function prediction, and can perform the best as our results show.

The MFR is fundamentally a regression model, including two kinds of core elements, features, and model. So, for the next step, we plan to improve the MFR model on these core elements. First, we will improve the MFR model through obtaining and using more available and more accurate prior knowledge, as the MFR has high accuracy and robustness, and its dependency on the prior-knowledge based similarities make it adaptable. Second, with the development of deep learning technology, recently more and more computational methods are constructed based on deep learning models. As deep learning models automatically learn the complex functions for mapping input features to output results, deep-learning-based methods achieve to state-of-the-art accuracy of many prediction tasks, including image recognition^88–90^ and natural language processing^91–93^. Therefore, we will use deep learning models, such as the deep belief network, to replace SVM for MFR to improve accuracy and robustness.

## Supplementary information

Using Machine Learning to Measure Relatedness Between Genes: A Multi-Features Model

## Data Availability

The trained MFR model is saved as an R data, and the datasets and the results of the current study can be freely downloaded at http://bmbl.sdstate.edu/MFR for academic uses, further verification, and biological analysis. The other data used and analyzed during the current study are available in this published article.

## References

[1] Du D, Rawat N, Deng Z, Gmitter GF (2015). Construction of citrus gene coexpression networks from microarray data using random matrix theory. Horticulture Research.

[2] Righetti, K. *et al*. Inference of Longevity-Related Genes from a Robust Coexpression Network of Seed Maturation Identifies Regulators Linking Seed Storability to Biotic Defense-Related Pathways. *Plant Cell* **27** (2015). 10.1105/tpc.15.00632 PMC4682330 26410298

[3] Sarkar NK, Kim YK, Grover A (2014). Coexpression network analysis associated with call of rice seedlings for encountering heat stress. Plant Molecular Biology.

[4] Takehisa H, Sato Y, Antonio B, Nagamura Y (2015). Coexpression Network Analysis of Macronutrient Deficiency Response Genes in Rice. Rice.

[5] Zhao X, Liu ZY, Liu QX (2015). Gene coexpression networks reveal key drivers of phenotypic divergence in porcine muscle. BMC Genomics.

[6] Beiki, H. *et al*. Large-scale gene co-expression network as a source of functional annotation for cattle genes. *Bmc Genomics* **17** (2016). 10.1186/s12864-016-3176-2 PMC5094014 27806696

[7] Wong DC, Sweetman C, Ford CM (2014). Annotation of gene function in citrus using gene expression information and co-expression networks. BMC Plant Biology.

[8] Yao P (2015). Coexpression networks identify brain region-specific enhancer RNAs in the human brain. Nature Neuroscience.

[9] Bulashevska S, Eils R (2005). Inferring genetic regulatory logic from expression data. Bioinformatics.

[10] Chen SC, Tsai TH, Chung CH, Li WH (2015). Dynamic association rules for gene expression data analysis. Bmc Genomics.

[11] Li, G., Ma, Q., Tang, H., Paterson, A. H. & Xu, Y. In *Nucleic Acids Research* (2009). 10.1093/nar/gkp491 PMC2731891 19509312

[12] Soinov LA, Krestyaninova MA, Brazma A (2003). Towards reconstruction of gene networks from expression data by supervised learning. Genome Biology.

[13] Stuart JM, Segal E, Koller D, Kim SK (2003). A gene-coexpression network for global discovery of conserved genetic modules. Science.

[14] Wolfe CJ, Kohane IS, Butte AJ (2005). Systematic survey reveals general applicability of “guilt-by-association” within gene coexpression networks. BMC Bioinformatics.

[15] Yu, Z. *et al*. QUBIC: a bioconductor package for qualitative biclustering analysis of gene co-expression data. *Bioinformatics* (2016). 10.1093/bioinformatics/btw635 28172469

[16] Eisen MB, Spellman PT, Brown PO, Botstein D, Botstein D (1998). Cluster analysis and display of genome-wide expression patterns. Proceedings of the National Academy of Sciences of the United States of America.

[17] Kotlyar M, Fuhrman S, Ableson A, Somogyi R (2002). Spearman Correlation Identifies Statistically Significant Gene Expression Clusters in Spinal Cord Development and Injury. Neurochemical Research.

[18] Basso K (2005). Reverse engineering of regulatory networks in human B cells. Nature Genetics.

[19] Carsten O Daub RS, Selbig J, Kloska S (2004). Estimating mutual information using B-spline functions – an improved similarity measure for analysing gene expression data. BMC Bioinformatics.

[20] Mehtiev AA (2006). ARACNE: An Algorithm for the Reconstruction of Gene Regulatory Networks in a Mammalian Cellular Context. BMC Bioinformatics.

[21] Steuer R, Kurths J, Daub CO, Weise J, Selbig J (2002). The mutual information: Detecting and evaluating dependencies between variables. Bioinformatics.

[22] Babak A, Frey BJ (2013). Network cleanup. Nature Biotechnology.

[23] Barzel B, Barabási AL (2013). Network link prediction by global silencing of indirect correlations. Nature Biotechnology.

[24] Feizi S, Marbach D, Médard M, Kellis M (2013). Network deconvolution as a general method to distinguish direct dependencies in networks. Nature Biotechnology.

[25] Zhang X (2012). Inferring gene regulatory networks from gene expression data by path consistency algorithm based on conditional mutual information. Bioinformatics.

[26] Okamura Y (2014). COXPRESdb in 2015: coexpression database for animal species by DNA-microarray and RNAseq-based expression data with multiple quality assessment systems. Nucleic Acids Research.

[27] Van DS, Craig T, de Magalhães JP (2014). GeneFriends: a human RNA-seq-based gene and transcript co-expression database. Nucleic Acids Research.

[28] Bass JIF (2013). Using networks to measure similarity between genes: association index selection. Nature Methods.

[29] Huang DW (2007). The DAVID Gene Functional Classification Tool: a novel biological module-centric algorithm to functionally analyze large gene lists. Genome Biology.

[30] Liu, W. *et al*. Gene Regulatory Networks from Gene Ontology. **7875**, 87–98 (2013).

[31] Harris MA (2004). The Gene Ontology (GO) database and informatics resource. Nucleic Acids Research.

[32] EM, Z. *et al*. OrthoDB v9.1: cataloging evolutionary and functional annotations for animal, fungal, plant, archaeal, bacterial and viral orthologs. *Nucleic acids research* (2016). 10.1093/nar/gkw1119 PMC5210582 27899580

[33] Kanehisa M (2002). The KEGG database. Novartis Foundation Symposium.

[34] Croft D (2011). Reactome: a database of reactions, pathways and biological processes. Nucleic Acids Research.

[35] D C (2014). The Reactome pathway knowledgebase. Nucleic Acids Research.

[36] Mishra GR (2006). Human protein reference database—2006 update. Nucleic Acids Research.

[37] Xenarios I (2002). DIP, the Database of Interacting Proteins: a research tool for studying cellular networks of protein interactions. Nucleic Acids Research.

[38] Bovolenta LA, Acencio ML, Lemke N (2012). HTRIdb: an open-access database for experimentally verified human transcriptional regulation interactions. BMC Genomics.

[39] Han H (2015). TRRUST: a reference database of human transcriptional regulatory interactions. Scientific Reports.

[40] Wang YX, Waterman MS, Huang H (2014). Gene coexpression measures in large heterogeneous samples using count statistics. Proceedings of the National Academy of Sciences of the United States of America.

[41] Song L, Langfelder P, Horvath S (2012). Comparison of co-expression measures: mutual information, correlation, and model based indices. Bmc Bioinformatics.

[42] Cortes C, Vapnik V (1995). Support-Vector Networks. Machine Learning.

[43] Pan XY, Zhang YN, Shen HB (2010). Large-Scale Prediction of Human Protein−Protein Interactions from Amino Acid Sequence Based on Latent Topic Features. Journal of Proteome Research.

[44] Venables WN, Ripley BD (2002). Modern Applied Statistics with S. Statistics & Computing.

[45] Obayashi T, Kinoshita K (2009). Rank of correlation coefficient as a comparable measure for biological significance of gene coexpression. DNA Research.

[46] Caragea C, Silvescu A, Mitra P (2012). Protein sequence classification using feature hashing. Proteome Science.

[47] Park Y, Marcotte EM (2012). Flaws in evaluation schemes for pair-input computational predictions. Nature Methods.

[48] Xue LC, Dobbs D, Honavar V (2011). HomPPI: a class of sequence homology based protein-protein interface prediction methods. BMC Bioinformatics.

[49] Blohm P (2013). Negatome 2.0: a database of non-interacting proteins derived by literature mining, manual annotation and protein structure analysis. Nucleic Acids Research.

[50] Barrett, T. *et al*. NCBI GEO: archive for functional genomics data sets—update. *Nucleic Acids Research* **41** (2013). 10.1093/nar/gks1193 PMC3531084 23193258

[51] Consortium UP (2016). UniProt: the universal protein knowledgebase. Nucleic Acids Research.

[52] Hampton T (2006). Cancer Genome Atlas. Journal of the American Medical Association.

[53] Lin, D. An information-theoretic measure of similarity. *Phase Noise Test Signal Generators Gigatronics Phase Noise Basics* (1998).

[54] Lifeng Chen DV (2006). Predicting genes for orphan metabolic activities using phylogenetic profiles. Genome Biology.

[55] Platt, J. C. *Fast training of support vector machines using sequential minimal optimization*. (MIT Press, 1999).

[56] Chang CC, Lin CJ (2011). LIBSVM: A library for support vector machines. Acm Transactions on Intelligent Systems & Technology.

[57] Meyer, P. E. Information-theoretic variable selection and network inference from microarray data. PhD thesis of the Universite Libre de Bruxelles (2008).

[58] Gelʹfand IM, Yaglom AM (2016). Calculation of the Amount of Information About a Random Function Contained in Another Such Function. Uspekhi Mat Nauk.

[59] Wyner AD (1978). A definition of conditional mutual information for arbitrary ensembles. Information & Control.

[60] Belhumeur PN, Hespanha JP, Kriegman D (1997). Eigenfaces vs. Fisherfaces: recognition using class specific linear projection. IEEE Transactions on Pattern Analysis and Machine Intelligence.

[61] Dudoit S, Fridlyand J, Speed TP (2011). Comparison of Discrimination Methods for the Classification of Tumors Using Gene Expression Data. Journal of the American Statistical Association.

[62] Guadagni PM, Little JDC (2008). A Logit Model of Brand Choice Calibrated on Scanner Data. Marketing Science.

[63] Nevo A (2000). A Practitioner’s Guide to Estimation of Random‐Coefficients Logit Models of Demand. Journal of Economics and Management Strategy.

[64] Hanley JA, Mcneil BJ (1982). The meaning and use of the area under a receiver operating characteristic (ROC) curve. Radiology.

[65] Fletcher RH, Fletcher SW, Wagner EH (2013). Clinical epidemiology: the essentials. Journal of the Royal College of General Practitioners.

[66] Zhou X, Kao MCJ, Wong WH (2002). Transitive functional annotation by shortest-path analysis of gene expression data. Proceedings of the National Academy of Sciences of the United States of America.

[67] Ritchie, M. E. *et al*. limma powers differential expression analyses for RNA-sequencing and microarray studies. *Nucleic Acids Research* **43** (2015). 10.1093/nar/gkv007 PMC4402510 25605792

[68] Newman MEJ (2003). Fast algorithm for detecting community structure in networks. Physical Review E.

[69] Newman MEJ, Girvan M (2003). Finding and evaluating community structure in networks. Physical Review E.

[70] Clauset A, Newman MEJ, Moore C (2004). Finding community structure in very large networks. Physical Review E.

[71] Benjamini Y, Yekutieli D (2001). The control of the false discovery rate in multiple testing under dependency. Annals of Statistics.

[72] Deberardinis RJ, Cheng T (2010). Q’s next: the diverse functions of glutamine in metabolism, cell biology and cancer. Journal of Accident & Emergency Medicine.

[73] Wise DR, Thompson CB (2010). Glutamine addiction: a new therapeutic target in cancer. Trends in Biochemical Sciences.

[74] Li H (2015). Long non-coding RNA UCA1 promotes glutamine metabolism by targeting miR-16 in human bladder cancer. Japanese Journal of Clinical Oncology.

[75] Friday E, Rd OR, Welbourne T, Turturro F (2011). Glutaminolysis and glycolysis regulation by troglitazone in breast cancer cells: Relationship to mitochondrial membrane potential. Journal of Cellular Physiology.

[76] Krall AS, Xu S, Graeber TG, Daniel B, Christofk HR (2016). Asparagine promotes cancer cell proliferation through use as an amino acid exchange factor. Nature Communications.

[77] Sodi, V. L. *et al*. mTOR/MYC Axis Regulates O-GlcNAc Transferase (OGT) Expression and O-GlcNAcylation in Breast Cancer. *Molecular Cancer Research Mcr* **13** (2015). 10.1158/1541-7786.MCR-14-0536 PMC4433402 25636967

[78] Suzuki S (2010). Phosphate-activated glutaminase (GLS2), a p53-inducible regulator of glutamine metabolism and reactive oxygen species. Proceedings of the National Academy of Sciences.

[79] Tedeschi PM (2012). Contribution of serine, folate and glycine metabolism to the ATP, NADPH and purine requirements of cancer cells. Cell Death & Disease.

[80] Thornburg JM (2008). Targeting aspartate aminotransferase in breast cancer. Breast Cancer Research.

[81] Todorova VK (2004). Effect of dietary glutamine on tumor glutathione levels and apoptosis-related proteins in DMBA-induced breast cancer of rats. Breast Cancer Research and Treatment.

[82] Iozzo RV, Clark CC (1987). Modulation of heparan sulfate biosynthesis. Effects of 6-diazo-5-oxo-L-norleucine and low glutamine on the synthesis of heparan sulfate proteoglycan by human colon carcinoma cells. Journal of Biological Chemistry.

[83] Hassanein M (2015). Targeting SLC1a5-mediated glutamine dependence in non-small cell lung cancer. Int J Cancer.

[84] Yang L (2014). Metabolic shifts toward glutamine regulate tumor growth, invasion and bioenergetics in ovarian cancer. Molecular Systems Biology.

[85] Yuan T (2017). Systematic analyses of glutamine and glutamate metabolisms across different cancer types. Chinese Journal of Cancer.

[86] DeBerardinis RJ (2007). Beyond aerobic glycolysis: transformed cells can engage in glutamine metabolism that exceeds the requirement for protein and nucleotide synthesis. Proceedings of the National Academy of Sciences.

[87] Sun L (2015). cMyc-mediated activation of serine biosynthesis pathway is critical for cancer progression under nutrient deprivation conditions. Cell Research.

[88] Lécun Y, Bottou L, Bengio Y, Haffner P (1998). Gradient-based learning applied to document recognition. Proceedings of the IEEE.

[89] Szegedy, C. *et al*. Going Deeper with Convolutions. 1–9 (2014).

[90] He, K., Zhang, X., Ren, S. & Sun, J. In *IEEE Conference on Computer Vision and Pattern Recognition*. 770–778.

[91] Bahdanau, D., Cho, K. & Bengio, Y. Neural Machine Translation by Jointly Learning to Align and Translate. *Computer Science* (2014).

[92] Cho, K. *et al*. Learning Phrase Representations using RNN Encoder-Decoder for Statistical Machine Translation. *Computer Science* (2014).

[93] Sak, H., Senior, A. & Beaufays, F. Long Short-Term Memory Based Recurrent Neural Network Architectures for Large Vocabulary Speech Recognition. *Computer Science*, 338–342 (2014).

