# Supplementary material for: Using Machine Learning to Measure Relatedness Between Genes: A Multi-Features Model
Source: Sci Rep. 2019 Mar 12;9:4192. doi: 10.1038/s41598-019-40780-7 (PMC6414665; doi:10.1038/s41598-019-40780-7)
Supplement: Supplementary file 1 — Using Machine Learning to Measure Relatedness Between Genes: A Multi-Features Model [file 41598_2019_40780_MOESM1_ESM.pdf]

# Using Machine Learning to Measure Relatedness Between Genes: A Multi-Features Model

Yan Wang[1], Sen Yang[1], Jing Zhao[2, 3], Wei Du[1], Yanchun Liang[1, 4], Cankun Wang[5], Fengfeng Zhou[1], Yuan Tian[1, 6] * and Qin Ma[5,7] *

[1] Key Laboratory of Symbol Computation and Knowledge Engineering of Ministry of Education, College of Computer Science and Technology, Jilin University, Changchun, 130012, China.
[2] Population Health Group, Sanford Research, Sioux Falls, SD, 57104, USA.
[3] Department of Internal Medicine, Sanford School of Medicine, University of South Dakota, Sioux Falls, SD, 57105, USA.
[4] Zhuhai Laboratory of Key Laboratory of Symbol Computation and Knowledge Engineering of Ministry of Education, Department of Computer Science and Technology, Zhuhai College of Jilin University, Zhuhai, 519041, China.
[5] Bioinformatics and Mathematical Biosciences Lab, Department of Agronomy, Horticulture, and Plant Science, Department of Mathematics and Statistics, South Dakota State University, Brookings, SD, 57006, USA.
[6] School of Artificial Intelligence, Jilin University, Changchun, 130012, China.
[7] Department of Biomedical Informatics, College of Medicine, The Ohio State University, Columbus, OH, 43210, USA.

*Corresponding authors: tianyuan12@mails.jlu.edu.cn; Qin.Ma@osumc.edu

| Network | Method | Pathway | Q-value | Module |
|---|---|---|---|---|
| 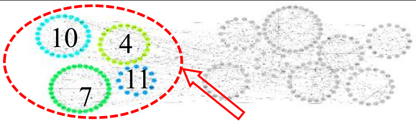  (A) Modules related with increased glutamine and glutamate metabolism by MFR | MFR | Glutathione metabolism | 3.27E-05 | 7, 10 |
| | MFR | Glycine, serine and threonine metabolism | 5.39E-04 | 4 |
| | MFR | Purine metabolism | 6.07E-04 | 11 |
| | MFR | Pyrimidine metabolism | 1.46E-13 | 10 |
| 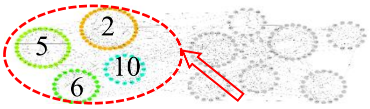  (B) Modules related with increased glutamine and glutamate metabolism by LDA | LDA | Glutathione metabolism | 9.52E-09 | 2 |
| | LDA | Glycine, serine and threonine metabolism | 4.72E-04 | 5 |
| | LDA | Purine metabolism | 8.64E-05 | 2 |
| | LDA | Pyrimidine metabolism | 1.64E-18 | 10 |
| 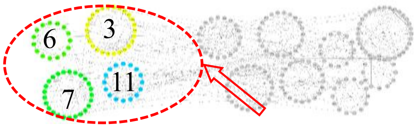  (C) Modules related with increased glutamine and glutamate metabolism by Logit | Logit | Glutathione metabolism | 2.27E-07 | 3 |
| | Logit | Glycine, serine and threonine metabolism | 4.71E-05 | 6 |
| | Logit | Purine metabolism | 5.00E-04 | 3 |
| | Logit | Pyrimidine metabolism | 1.64E-18 | 11 |
| 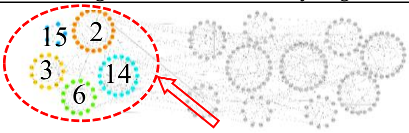  (D) Modules related with increased glutamine and glutamate metabolism by PCC | PCC | Purine metabolism | 7.00E-05 | 6 |
| | PCC | Pyrimidine metabolism | 1.20E-22 | 14 |
| 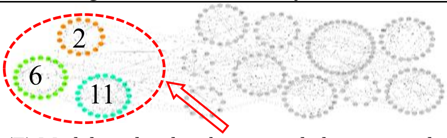  (E) Modules related with increased glutamine and glutamate metabolism by SRC | SRC | Glycine, serine and threonine metabolism | 3.21E-05 | 6 |
| | SRC | Pyrimidine metabolism | 2.54E-19 | 6 |
| 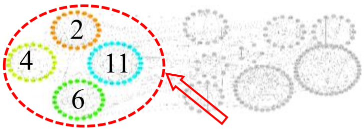  (F) Modules related with increased glutamine and glutamate metabolism by PPC | PPC | Glycine, serine and threonine metabolism | 2.10E-04 | 11 |
| | PPC | Purine metabolism | 4.84E-04 | 2 |
| | PPC | Glutathione metabolism | 1.50E-04 | 6 |
| 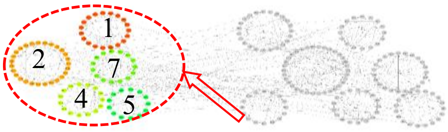  (G) Modules related with increased glutamine and glutamate metabolism by MI | MI | Glutathione metabolism | 2.25E-05 | 2 |
| | MI | Pyrimidine metabolism | 8.99E-20 | 1 |
| | MI | Purine metabolism | 1.56E-04 | 5 |
| 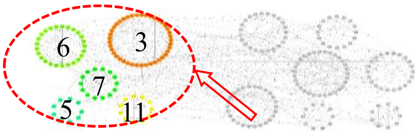  (H) Modules related with increased glutamine and glutamate metabolism by CMI | CMI | Glutathione metabolism | 1.27E-04 | 7 |
| | CMI | Pyrimidine metabolism | 1.40E-04 | 11 |
| 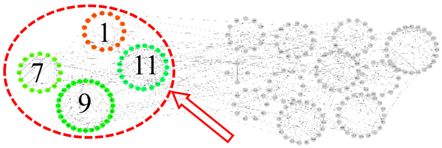  (I) Modules related with increased glutamine and glutamate metabolism by CXP | CXP | Pyrimidine metabolism | 2.72E-19 | 9 |
| | CXP | Purine metabolism | 2.88E-04 | 11 |

**Figure S1.** Metabolic pathways are predicted to be directly influenced by increased glutamine and glutamate metabolism in nine BLCA gene networks

| Network | Method | Pathway | Q-value | Module |
|---|---|---|---|---|
| 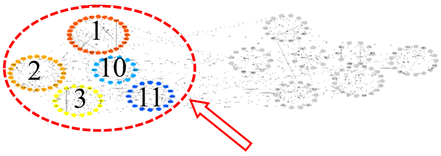 (A) Modules related with increased glutamine and glutamate metabolism by MFR | MFR | Glycine, serine and threonine metabolism | 1.75E-04 | 2 |
| | MFR | Purine metabolism | 2.15E-08 | 2, 10 |
| | MFR | Pyrimidine metabolism | 2.72E-31 | 1, 3, 11 |
| 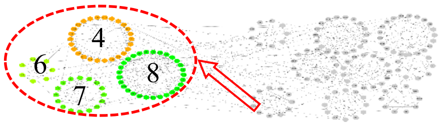 (B) Modules related with increased glutamine and glutamate metabolism by LDA | LDA | Glycine, serine and threonine metabolism | 7.17E-09 | 6 |
| | LDA | Purine metabolism | 6.69E-21 | 4, 8 |
| | LDA | Pyrimidine metabolism | 2.00E-21 | 4, 7, 8 |
| 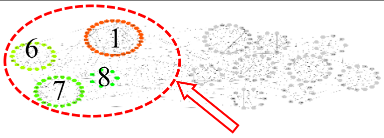 (C) Modules related with increased glutamine and glutamate metabolism by Logit | Logit | Glycine, serine and threonine metabolism | 7.17E-09 | 8 |
| | Logit | Purine metabolism | 2.70E-20 | 1, 7 |
| | Logit | Pyrimidine metabolism | 7.24E-21 | 1, 6, 7 |
| 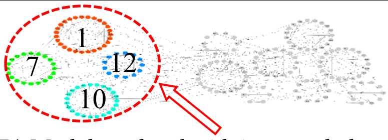 (D) Modules related with increased glutamine and glutamate metabolism by PCC | PCC | Glycine, serine and threonine metabolism | 1.49E-04 | 1 |
| | PCC | Pyrimidine metabolism | 3.68E-13 | 1, 7 |
| | PCC | Purine metabolism | 2.04E-11 | 1, 7 |
| 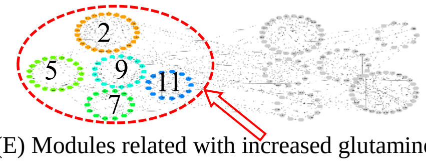 (E) Modules related with increased glutamine and glutamate metabolism by SRC | SRC | Glycine, serine and threonine metabolism | 3.14E-05 | 7 |
| | SRC | Pyrimidine metabolism | 3.61E-04 | 5, 9 |
| | SRC | Purine metabolism | 4.55E-13 | 7, 9 |
| 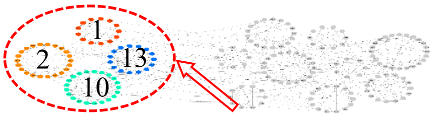 (F) Modules related with increased glutamine and glutamate metabolism by PPC | PPC | Glycine, serine and threonine metabolism | 1.68E-08 | 10 |
| | PPC | Purine metabolism | 1.16E-10 | 1, 10 |
| | PPC | Pyrimidine metabolism | 2.00E-04 | 2 |
| 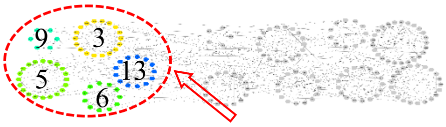 (G) Modules related with increased glutamine and glutamate metabolism by MI | MI | Glycine, serine and threonine metabolism | 1.43E-05 | 6 |
| | MI | Pyrimidine metabolism | 3.30E-05 | 3 |
| | MI | Purine metabolism | 2.41E-07 | 3 |
| 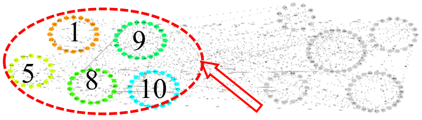 (H) Modules related with increased glutamine and glutamate metabolism by CMI | CMI | Pyrimidine metabolism | 1.53E-10 | 10 |
| | CMI | Purine metabolism | 1.53E-10 | 5, 10 |
| 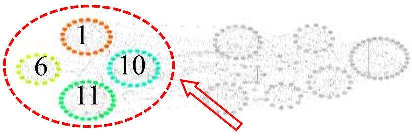 (I) Modules related with increased glutamine and glutamate metabolism by CXP | CXP | Glycine, serine and threonine metabolism | 8.67E-05 | 11 |
| | CXP | Purine metabolism | 1.99E-13 | 10, 11 |
| | CXP | Pyrimidine metabolism | 4.39E-14 | 11 |

**Figure S2.** Metabolic pathways are predicted to be directly influenced by increased glutamine and glutamate metabolism in nine COAD gene networks.

| Network | Method | Pathway | Q-value | Module |
|---|---|---|---|---|
| 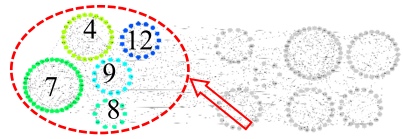  (A) Modules related with increased glutamine and glutamate metabolism by MFR | MFR | Amino sugar and nucleotide, sugar metabolism | 7.72E-08 | 8 |
| | MFR | Glutathione metabolism | 2.45E-11 | 4, 12 |
| | MFR | Glycine, serine and threonine metabolism | 1.31E-07 | 7 |
| | MFR | Purine metabolism | 8.33E-08 | 4 |
| | MFR | Pyrimidine metabolism | 6.95E-12 | 7 |
| 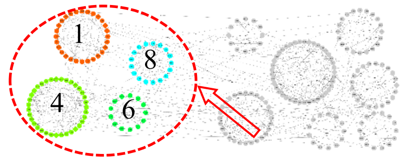  (B) Modules related with increased glutamine and glutamate metabolism by LDA | LDA | Amino sugar and nucleotide sugar metabolism | 1.66E-11 | 1 |
| | LDA | Glutathione metabolism | 7.80E-11 | 6, 8 |
| | LDA | Glycine, serine and threonine metabolism | 2.19E-04 | 4 |
| | LDA | Purine metabolism | 1.24E-10 | 6 |
| | LDA | Pyrimidine metabolism | 6.36E-36 | 4 |
| 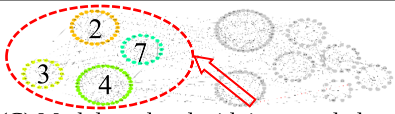  (C) Modules related with increased glutamine and glutamate metabolism by Logit | Logit | Glutathione metabolism | 2.01E-10 | 2, 7 |
| | Logit | Glycine, serine and threonine metabolism | 3.00E-06 | 4 |
| | Logit | Purine metabolism | 8.00E-07 | 2 |
| | Logit | Pyrimidine metabolism | 1.18E-33 | 4 |
| 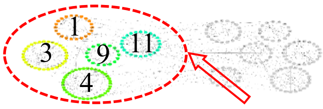  (D) Modules related with increased glutamine and glutamate metabolism by PCC | PCC | Glutathione metabolism | 1.18E-09 | 11 |
| | PCC | Purine metabolism | 4.99E-18 | 3 |
| | PCC | Pyrimidine metabolism | 9.65E-21 | 3 |
| 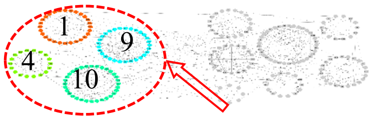  (E) Modules related with increased glutamine and glutamate metabolism by SRC | SRC | Glutathione metabolism | 6.06E-13 | 9 |
| | SRC | Purine metabolism | 1.36E-16 | 10 |
| | SRC | Pyrimidine metabolism | 4.62E-19 | 1, 10 |
| 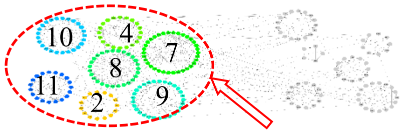  (F) Modules related with increased glutamine and glutamate metabolism by PPC | PPC | Glutathione metabolism | 7.92E-13 | 11 |
| | PPC | Glycine, serine and threonine metabolism | 3.24E-06 | 4 |
| | PPC | Purine metabolism | 1.12E-07 | 8 |
| | PPC | Pyrimidine metabolism | 9.46E-18 | 10 |
| 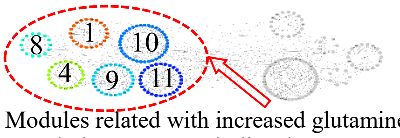  (G) Modules related with increased glutamine and glutamate metabolism by MI | MI | Glutathione metabolism | 1.43E-12 | 4 |
| | MI | Purine metabolism | 6.42E-15 | 11 |
| | MI | Pyrimidine metabolism | 2.63E-19 | 11 |
| 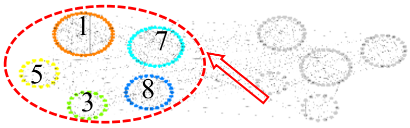  (H) Modules related with increased glutamine and glutamate metabolism by CMI | CMI | Purine metabolism | 3.22E-06 | 1 |
| | CMI | Glycine, serine and threonine metabolism | 2.22E-04 | 3 |
| | CMI | Glutathione metabolism | 1.50E-05 | 3 |
| 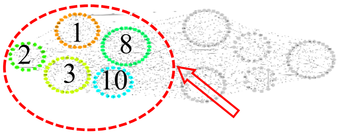  (I) Modules related with increased glutamine and glutamate metabolism by CXP | CXP | Glutathione metabolism | 1.46E-11 | 10 |
| | CXP | Purine metabolism | 4.07E-14 | 1 |
| | CXP | Pyrimidine metabolism | 1.44E-16 | 1 |

**Figure S3.** Metabolic pathways are predicted to be directly influenced by increased glutamine and glutamate metabolism in nine LUAD gene networks
